# Fat Pulmonary Embolism With Crazy-Paving Pattern Opacities and Pneumothorax: A Rare Complication of Liposuction

**DOI:** 10.7759/cureus.40607

**Published:** 2023-06-18

**Authors:** Yusra Ansari, Saad Ali Ansari, Maryam Hussain, Nisar Kazimuddin, Tahir Muhammad Abdullah Khan

**Affiliations:** 1 Internal Medicine, University of Kentucky College of Medicine, Bowling Green, USA; 2 Internal Medicine, University of California Riverside School of Medicine, Riverside, USA; 3 Internal Medicine, Crozer-Chester Medical Center, Upland, USA; 4 Pulmonary and Critical Care Medicine, Med Center Health, Bowling Green, USA; 5 Pulmonary and Critical Care, University of Kentucky College of Medicine, Bowling Green, USA

**Keywords:** crazy paving pattern, pneumothorax (ptx), acute hypoxic respiratory failure, pulmonary fat embolism, liposuction complication

## Abstract

Fat embolism syndrome (FES) is a rare multiorgan disease caused by microvascular obstruction by fat globules and free fatty acid-mediated endothelial injury leading to pro-inflammatory cytokine release. We present a rare case of a 54-year-old woman who underwent elective aesthetic liposuction and developed FES and pneumothorax within 12 hours of the procedure.

## Introduction

Fat embolism syndrome (FES) is commonly associated with long bone fractures and orthopedic procedures, but due to the increasing number of aesthetic liposuction procedures, more cases of FES are being reported. Fat pulmonary embolism (FPE) can manifest as acute hypoxic respiratory failure, requiring mechanical ventilation support. It can also lead to right heart strain and cardiac arrest if there is a significant burden of fat embolism. Other features of FES may include a petechial rash, confusion, fever, and tachycardia [1-3]. Pneumothorax following liposuction is another infrequent complication associated with liposuction with an incidence of 0.0432%, however, it is more common with axillary liposuction [4]. We report a rare complication of hypoxic respiratory failure with pneumothorax and diffuse crazy-paving imaging pattern due to FPE after an abdominal liposuction procedure.

## Case presentation

**Figure 1:**
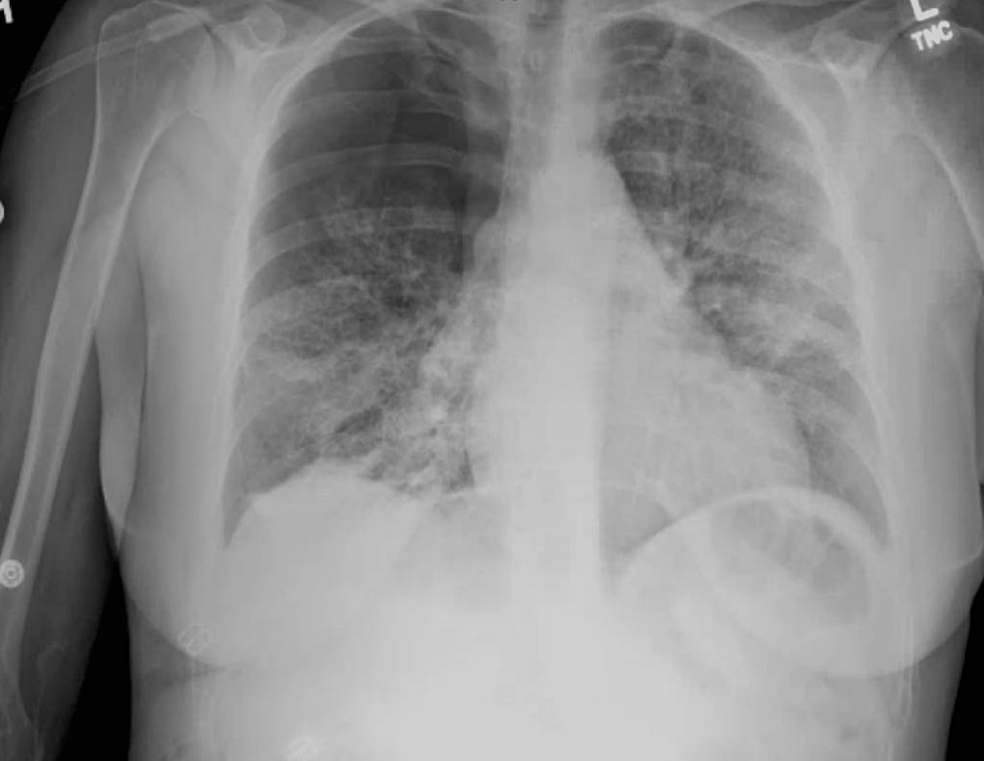
Chest x-ray showing right-sided pneumothorax and diffuse infiltrates.

A 54-year-old woman with a history of endometriosis, anxiety, depression, and active tobacco use (with a 40-pack-year history) underwent elective abdominal aesthetic liposuction. The patient was discharged home after the procedure without any immediate complications. Within 12 hours of the procedure, the patient developed shortness of breath at rest associated with a dry cough and subjective fevers without chills. The patient denied chest pain, hemoptysis, nausea, vomiting, or other gastrointestinal complaints. Due to the progression of breathing difficulty, the patient presented to the emergency department and was found to be tachypneic and tachycardiac, with pulse oximetry showing 80% oxygen saturation on room air. Vital signs were a heart rate of 112 per minute with sinus rhythm, a respiratory rate of 35 per minute without accessory respiratory muscle use, a temperature of 99.2^o^F, and a blood pressure of 132/80 mmHg. The patient appeared short of breath on examination, and bilateral diffuse crackles were audible on auscultation. The patient had sinus tachycardia without any abnormal heart sounds. The rest of the physical examination was normal. Lab work showed a white blood cell count of 19 k/µL (4-11 k/µL) with 77.5% neutrophils, 11.7% lymphocytes, and 0.1% eosinophils. Hemoglobin was 11.5 g/dl (12-15.5 g/dl), and the platelet count was 286 k/µL (150-400 k/µL). Arterial blood gas showed PH 7.42, partial pressure of oxygen (pO_2_) 72 mm Hg, partial pressure of carbon dioxide (pCO_2_) 33 mmHg, on supplemental oxygen support via high flow nasal cannula with fraction of inspired oxygen (FiO_2_) of 1, and arterial oxygen pressure (PaO_2_)/FiO_2_ ratio was 72. C-reactive protein was 23 mg/dl (less than 0.9 mg/dl), procalcitonin was 0.1 ng/ml (less than 0.1 ng/ml), serum creatinine was 0.57 mg/dl (0.52-1.04), Alanine transaminase (ALT) was 56 U/L (0-34), Aspartate transaminase (AST) was 47 U/L (14-36), and alkaline phosphatase (ALP) was 151 U/L (38-126). Serum lactate dehydrogenase was normal. Electrolytes were normal. Chest X-ray showed bilateral diffuse patchy airspace disease and right pneumothorax (Figure 1).

**Figure 2:**
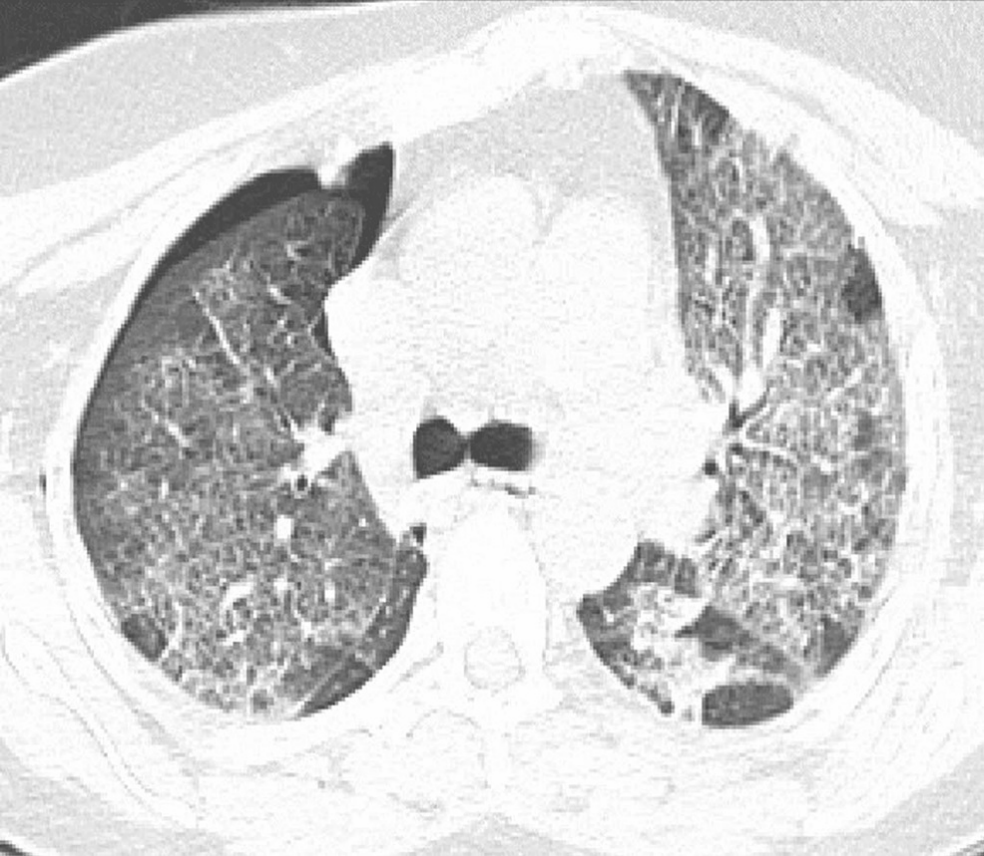
Chest CT showing crazy-paving pattern of bilateral diffuse ground glass infiltrates with smooth interlobular septal thickening and right-sided pneumothorax.

A 28 Fr chest tube was placed on the right side with the resolution of pneumothorax, and subsequent chest CT showed bilateral diffuse ground-glass infiltrates with smooth interlobular septal thickening, suggestive of a crazy-paving pattern. A small residual anterior right upper pneumothorax was observed with the chest tube positioned under the base of the right lung (Figure 2). 

The chest tube was placed under negative 20 cm H_2_O suction, and empiric broad antibiotics with vancomycin and cefepime were initiated after sputum and blood cultures were drawn. Over the next 48 hours, the patient continued to require high-flow supplemental oxygen with a mild decrease in oxygen requirement to 0.86 FiO_2_ and remained afebrile and hemodynamically stable. The sputum BioFire® FilmArray® Pneumonia polymerase chain reaction (PCR) panel and respiratory viral panel (BioFire Diagnostics, Salt Lake City, Utah, United States) showed no bacterial or viral microorganisms. Later, no microbial growth was observed on sputum and blood cultures, and repeat procalcitonin after 48 hours remained normal, i.e., 0.1 ng/ml. After three days of admission, the patient started to improve clinically with decreasing oxygen requirement, and inflammatory markers, including leukocyte count, started to trend down. Although the patient was offered bronchoscopy and bronchoalveolar lavage examination for diagnostic purposes, she declined as she continued to improve with supportive care. The patient did not have any active air leak from the right chest cavity for two days and the chest x-ray showed a resolution of pneumothorax. Therefore, the chest tube was removed without any complications.

Considering the temporal association of the onset of respiratory symptoms with a liposuction procedure, non-toxic appearance, normal procalcitonin, and negative infectious workup, the diagnosis of acute FPE was considered, and the antibiotics were discontinued. The patient was provided supportive care with supplemental oxygen, deep venous prophylaxis with enoxaparin, incentive spirometry, and encouraged ambulation. No systemic steroids or therapeutic anticoagulants were given. The patient manifested clinical and radiological improvement over eight days and was discharged home on 2 L per minute nasal cannula supplemental oxygen. At the outpatient follow-up visit two weeks after discharge, the patient was asymptomatic and did not require supplemental oxygen, and the repeat chest X-ray showed complete resolution of pneumothorax and near-complete resolution of bilateral parenchymal infiltrates with mild basilar atelectasis. 

## Discussion

FES is a rare condition that is diagnosed when fat globules are observed in the pulmonary venous circulation, with or without clinical symptoms. FES commonly occurs in settings of orthopedic trauma, with a higher incidence in closed, long bone fractures of the lower extremities. However, non-orthopedic causes include pancreatitis, bone marrow transplant, sickle cell crisis with bone marrow infarction, alcoholic liver disease, and rarely, liposuction [1,2].

FES is a rare condition that is predominantly diagnosed clinically. There is no definitive test to diagnose FES, requiring clinical correlation of symptoms with imaging and lab findings. FES can be a challenging diagnosis as its presentation can mimic other causes of respiratory distress in post-trauma and post-surgical patients. Gurd's and Wilson's criteria (Table 1) and Schonfeld's criteria (Table 2) are two scoring models that can help with the diagnosis [5]. Gurd's and Wilson's criteria consist of major and minor criteria, with a positive result obtained when at least one major and four minor criteria are met. Schonfeld's criteria consist of seven criteria with 16 points, and a diagnosis is reached when at least five points are met. Our patient met Schonfeld's criteria with a score of 8; i.e., alveolar infiltrates on chest radiograph (4), hypoxemia (3), and a respiratory rate > 30 breaths per minute (1). The patient did have low-grade fevers and tachycardia, but this did not meet the threshold cutoff of Schonfeld's and Gurd's, and Wilson's criteria. 

**Table 1:** Gurd's and Wilson’s criteria for the diagnosis of fat pulmonary embolism ESR: erythrocyte sedimentation rate

Major criteria	Minor criteria
Hypoxia (<60 mmHg O_2_)	Pyrexia (>39 °C)
Confusion	Tachycardia (>120 beats per minute)
Petechial rash	Retinal changes (petechiae)
	Anuria or oliguria
	Anemia (hemoglobin drop by 20%)
	Thrombocytopenia (drop by 50%)
	High ESR ( >71 mm per hour)
	Fat macroglobulinemia

**Table 2:** Schonfeld’s criteria for the diagnosis of fat pulmonary embolism

Symptoms	Points
Petechial Rash	5
Alveolar infiltrates on chest radiograph	4
Hypoxemia (<70 mmHg)	3
Confusion	1
Fever > 38 °C	1
Heat rate >120 beats per minute	1
Respiratory rate >30 breaths per minute	1

Imaging findings can be helpful in establishing a diagnosis. For instance, a chest X-ray may show bilateral diffuse or patchy infiltrates, but the results are generally non-specific and unable to distinguish it from other causes of respiratory distress. CT imaging is usually more helpful, and the findings can include airspace consolidations in severe cases or bilateral patchy ground-glass opacities with associated interlobular septal thickening, also known as the crazy-paving pattern [3,6]. Our patient had similar CT imaging findings. It's essential to note that the crazy-paving pattern can be seen with multiple causes, including pulmonary edema, acute respiratory distress syndrome (ARDS), pulmonary alveolar proteinosis (PAP), acute interstitial pneumonia (AIP), pulmonary hemorrhage, invasive mucinous adenocarcinoma of the lung, alveolar sarcoidosis, lipoid pneumonia, and pulmonary infections due to bacterial or fungal agents [6]. Therefore, a thorough workup, including bronchoscopy and bronchoalveolar lavage, is necessary before conclusively attributing a crazy-paving pattern to FPE.

Pneumothorax is an infrequent complication of liposuction, which has been reported in the literature with an incidence of 0.0432%, according to one study [4]. Potential risk factors include liposuction of the axilla, the use of flexible infiltration cannulas, and scarring from previous liposuction [4]. It is noteworthy that our patient developed pneumothorax during the liposuction of the abdomen, but the potential for intrathoracic injury still exists when the liposuction cannula is passed in a cephalad direction over the costal margin. Moreover, it should be noted that the patient had a significant history of tobacco smoking, which can predispose to secondary pneumothorax if there is an underlying undiagnosed emphysematous lung disease. No pulmonary clearance was sought before the procedure, which may have helped identify such a condition. 

FES following liposuction is rare, with only a few cases described in the literature [5]. It is possible that small blood vessels may rupture with damaged adipocytes during liposuction, producing lipid microfragments that enter the venous circulation and subsequently cause lung damage. The exact pathogenesis is not well elucidated, but it is proposed that the fat microglobules can lead to injury by two mechanisms: mechanical obstruction of small vessels and biochemical injury.

Firstly, fat microglobules induce a proinflammatory and prothrombotic state leading to platelet aggregation and fibrin generation, which can cause pulmonary microcirculation occlusion/thrombosis. Pulmonary capillary microthrombosis can lead to interstitial hemorrhage, edema, and hypoxic vasoconstriction [7]. Large-volume fat droplets can lead to massive embolism with right ventricular strain and circulatory shock. Secondly, biochemical injury may occur when tissue lipases break down fat into glycerol and toxic free fatty acids, which cause toxic injury to the pneumocytes and pulmonary endothelial cells and release a proinflammatory cytokine cascade, including tumor necrosis factor-alpha, interleukin-1, and interleukin-6. These inflammatory cytokines cause acute lung injury resulting in pulmonary edema and hemorrhage [7-9]. Furthermore, C-reactive protein is frequently elevated in FES, and it can also promote lipid agglutination, resulting in microvascular obstruction [8,9]. This biochemical theory of injury explains why liposuction-associated FES usually occurs within 12-72 hours following surgery [5].

There is no definitive treatment for FES, and management predominantly consists of supportive measures, with a mortality rate of 0.7-1.2% reported in a study by Bederman et al. [10]. Patients with severe hypoxic respiratory failure may require mechanical ventilation support. If massive fat embolism occurs, leading to right ventricular strain and circulatory collapse, the patient will need hemodynamic support with vasopressor and inotropic agents. In refractory cases, extracorporeal membrane oxygenation and circulatory support may be required.

Corticosteroids are often used in cases of severe FES, which may reduce hypoxia, but they have not shown a mortality benefit in patients with FES [10]. Although prophylactic administration of corticosteroids in patients undergoing surgery for long bone fractures has been suggested to prevent FES, a large randomized clinical trial is needed to prove its protective effect [10]. Therapeutic anticoagulation has no proven beneficial role in managing FES. In a few case reports, a possible protective role of N-acetylcysteine and human albumin infusion, especially in patients with FES and hypoalbuminemia, has been suggested, but it is not proven [11]. Our patient improved with supportive care alone without any anticoagulation or steroid use. 

## Conclusions

FPE and pneumothorax can be rare complications of the liposuction procedure and should be suspected in any patient who develops hypoxia within 12-48 hours following liposuction. Patients should be informed of these possible complications associated with the procedure and ensure post-surgical monitoring and follow-up to promptly detect and manage any complications to reduce morbidity and mortality.
